# Complexation of fish skin gelatin with glutentin and its effect on the properties of wheat dough and bread

**DOI:** 10.1016/j.fochx.2022.100319

**Published:** 2022-04-28

**Authors:** Shangyuan Sang, Changrong Ou, Yaqian Fu, Xueqian Su, Yamei Jin, Xueming Xu

**Affiliations:** a Key Laboratory of Animal Protein Food Deep Processing Technology of Zhejiang Province, College of Food and Pharmaceutical Sciences, Ningbo University, Ningbo, Zhejiang 315832, China; b Department of Food Science and Technology, Virginia Polytechnic Institute and State University, Blacksburg, VA 24061, United States; c The State Key Laboratory of Food Science and Technology, School of Food Science and Technology, Jiangnan University, Wuxi, Jiangsu 214122, China

**Keywords:** GEL3, amino acid repetitive sequence [Gly-Pro-Hyp]_6_ model in a gelatin segment, GLU3, amino acid repetitive sequence [GQQ]_6_ model in a glutenin segment, GLU6, amino acid repetitive sequence [PGQGQQ]_3_ model in a glutenin segment, GLU9, amino acid repetitive sequence [GYYPTSPQQ]_2_ model in a glutenin segment, WF, wheat flour, Fish skin gelatin, Dough, Complexation, Molecular dynamics simulation, Water diffusion, Crumb firming

## Abstract

This study aimed to investigate the effect of fish skin gelatin (*Gadus morhua*, 0.5%, or 1.0%, flour basis) on the properties of wheat (*Triticum aestivum*) dough and bread. Compared with the control group, the addition of 1.0% gelatin increased the storage modulus and the maximum resistance of dough, resulting in a longer rupture time and a larger final gas-retention volume of the dough. Bread characteristics showed that the specific loaf volume and crumb cell size both increased. Molecular dynamics simulation indicated that gelatin and glutenin segments formed a complex, where a large amount of hydroxyl groups on the surface retarded water mobility in bread. Gelatin-glutentin complexes with the high water-holding capacity inhibited water diffusion from marginal crumb to crust, and decreased starch retrogradation enthalpy and firming rate of crumb. Thus, fish skin gelatin might be a good improver of wheat dough and bread.

## Highlights

• A complex of fishskin gelatin and glutenin was formed in dough.
• Gelatin enhanced the strength and gas-retention capacity of dough.
• Gelatin improved the porosity and cell size of crumb and the specific volume of bread.
• Gelatin retarded the firming rate of crumb and starch retrogradation.
• Gelatin inhibited water diffusion from marginal crumb to crust.

## Introduction

High-quality bread properties, e.g., large volume, golden and crispy crust, soft and resilient crumb, and long shelf life, are related to the nature of wheat flour (WF), dough properties and bread processing. The bread quality heavily depends on the technological functionality of gluten proteins (Delcour et al., 2012). The glutenin subunits are cross-linked through both noncovalent bonds and covalent disulfide (SS) to form a gluten network in dough (Shewry & Halford, 2002). Consequently, dough improvers have long been used to enhance covalent crosslinking, often oxidative reagents (Liang, Gao, Yu, & Yang, 2021), or noncovalent binding, like hydrocolloids between glutenin subunits. Because of the public health concerns, hydrocolloids are usually recognized as an acceptable dough improver. In addition, the maintenance of desirable baking qualities during storage, and consequently, an extended shelf life, is always preferable for freshly-baked bread. However, staling would inevitably occur in bread products after long-term storage, especially under adverse environmental conditions. The crust staling is generally caused by water diffusion from crumb to crust, leading to a soft and leathery texture. On the contrary, the crumb-staling (or firming) process is partially associated with amylopectin retrogradation/recrystallization (Gray & Bemiller, 2003).

In addition to the well-known role of hydrocolloids (including polysaccharides and proteins) as dough improvers, their excellent staling inhibition properties proved in recent studies make them superior anti-staling agents for bread products (Ferrero, 2017). Some polysaccharide-based hydrocolloids (carrageenan isoform and pectin) can form hydrophilic complexes with gluten proteins to strengthen the dough and thus increase the loaf volume of bread (Ribotta, Ausar, Beltramo, & León, 2005). They usually have an anti-firming impact on the bread crumb by inhibiting the water mobility during storage due to the high water-retention capacity of hydrocolloids (Guarda, Rosell, Benedito, & Galotto, 2004). Regarding protein hydrocolloids, a larger elasticity of the dough as well as a lower firming rate in the final bread upon storage were observed with the addition of pigskin gelatin (Yu et al., 2019). However, the application of gelatins from mammalian sources (mostly porcine and bovine resources) was more or less restricted in recent years because of the vegetarian, halal and kosher markets (Karim & Bhat, 2008). As a result, the industrial use of collagen or gelatin obtained from non-mammalian species is now growing in importance. Fish gelatin may be a better alternative to mammalian gelatins (Karim & Bhat, 2009). It has good technological and nutritional functions in many food categories, such as gelation, emulsification, and skin health promotion (Gómez-Guillén et al., 2011, Liu et al., 2015). The melting temperature of gel made from fish gelatin is about 11 °C–26 °C, lower than that of pigskin gelatin (31 °C–36 °C) (Duconseille et al., 2018, Karim and Bhat, 2009). This means that the skin gelatin from cold-water fish can be dissolved in water at room temperature (20 °C–25 °C). Li, Zhang, Zhao, Ding, and Lin (2018) found that skin gelatin from cold-water fish could be complexed with Arabic gum driven by negative enthalpy owing to the electrostatic interaction and hydrogen bonds. Thus, it can be hypothesized that skin gelatin from cold-water fish can entangle with the central segment of glutenin molecule at room temperature during dough mixing to strengthen the dough. Meanwhile, the high swelling and water-binding capacity of solubilized gelatin makes them favorable materials for reducing water mobility to retard bread staling. Therefore, fish skin gelatin may be a promising ingredient for the production of bread with good sensory quality and health-promoting benefits.

## Materials and methods

### Materials

Dry yeast (*Saccharomyces cerevisiae*, Hubei Angel Yeast Co., Ltd., Wuhan, Hubei, China) and soft bread wheat flour (*Triticum aestivum*) containing 72.0% starch, 12.8% protein and 11.2% moisture on a dry matter basis (Yihai-Kerry Co., Ltd., Suzhou, Jiangsu, China) were used for bread-making. White granulated sugar (Guangxi Dongmen Nanhua Co., Ltd., Congzuo, Guangxi, China) is a commercial food-grade product. Fish (*Gadus morhua*) skin gelatin (purity 89%, average molecular weight ∼ 70 kDa) was purchased from Sigma–Aldrich Co., Ltd. (Shanghai, China). H_2_SO_4_ were purchased from China National Pharmaceutical Group Co., Ltd. (Shanghai, China).

### Extension tests of dough

The wheat dough without yeast was composed of WF (100%), distilled water (62.0% w/w, based on WF), sugar (6.0% w/w, on WF), and fish skin gelatin (0.0%, 0.5%, and 1.0%, separately, w/w, on WF, named as dough-0.0%, dough-0.5%, and dough-1.0%, respectively). The sugar and gelatin ingredients were dissolved in water before they were mixed with flour in a mixer (Sinmag Mechanical Co., Ltd., Wuxi, Jiangsu, China) at a low speed for 5 min, and then at high speed for 10 min until these developed doughs could be expanded into a thin continuous membrane. Extension tests were performed as previously described (Wang et al., 2019). The developed dough was first flattened into sheets with a thickness of 6 mm. Then, the sheets were cut into strips with a length of 190 mm and a width of 6 mm. These strips (190 × 6 × 6 mm^3^) were covered with a small amount of oil before they were extended by a texture analyzer. Dough extension curves were obtained using a TA.XT Plus Texture Analyzer with a Kieffer extensibility rig (Stable Micro System Co. Ltd., Surrey, England). In the tension mode, the dough strips were stretched from 20 to 160 mm at a speed of 3.30 mm/s. The force (mN) versus distance (mm) curves were measured, which ranged from 0 to 140 mm. At the maximum force point, the force (mN) and distance (mm) were, respectively, noted as resistance (mN) and extension (mm), which were used as an indication of dough extensibility.

### Oscillatory shearing tests of dough

The dough developed without yeast was analyzed using a DHR-3 rheometer equipped with a 20-mm parallel plate (TA Instrument, New Castle, DE, US) as previously described with modification (Primacella, Wang, & Acevedo, 2018). Oscillation strains in the range of 0.001%–1.000% were applied within a 2.000-mm gap at a frequency of 1.0 Hz and 15 points per decade. In the curve of storage modulus vs. oscillation strain, the average storage modulus (G') within the linear viscoelastic region was recognized as a measure of dough strength. In the data table of storage modulus vs oscillation stress measured by the rheometer, yield stress (σ*) was obtained as oscillation stress where the G' reached a value of 90% average G' within linear region.

### Gas-producing and expansion volume of dough

The dough developed with yeast (1.5% w/w, on WF) was prepared by the previous method in Section 2.2. Gas-producing and expansion volume were determined by the method described by Sang et al. (2020). The gassing power was measured by quantifying the released CO_2_ volume. The developed dough was split into 50 g each, and put in a sterilized vessel with a rubber plug and hose, which was previously heated in a water bath at 37 °C for 5 min. After the plug was loaded, CO_2_ was diffused through the hose into the inverted graduated cylinder filled with an acidic solution (H_2_SO_4_, 5 mmol/L, pH 2). The liquid was gradually replaced by CO_2_ in the graduated cylinder, where the CO_2_ volume can be measured. The measurement time was 70 min and the total produced CO_2_ volume was defined as the gas-producing volume of the dough.

The dough expansion occurred during the fermentation process. The dough pieces (50 g) were placed at the bottom of a sterilized graduated cylinder (250 mL). Then, they were fermented at 37 °C with 80% relative humidity for 70 min. The increase in the dough volume (mL) was calculated as the dough expansion volume. All the measurements were performed in triplicate.

### Molecular dynamics simulation

The amino acid sequence of fish skin gelatin consists of Gly-Pro-Hyp repeating triplets (S.-K. Kim, Y.-T. Kim, Byun, Nam, Joo, & Shahidi, 2001). In the central domain of the glutenin subunit, amino acid sequence contains three repetitive units, including tri (GQQ), hexa (PGQGQQ), and nonapeptide (GYYPTSPQQ) (Anderson and Greene, 1989, Shewry et al., 1992). Therefore, segments of gelatin or glutenin repetitive domain consisting of 18 amino acid residues (18-mer, GEL3: [Gly-Pro-Hyp]_6_, GLU3: [GQQ]_6_, GLU6: [PGQGQQ]_3_, GLU9: [GYYPTSPQQ]_2_) were employed in all simulations. The initial structure of the 18-mer gelatin or glutenin segments was set up using a tleap program in the AmberTools18 software. These built peptides were all capped by an acetyl group (CH_3_CO–) at *N*-terminal and a methylamino group (CH_3_NH–) at C-terminal, because they are protein segments. The initial distance between the center of one 18-mer gelatin and one glutenin segment was 2 nm using the Visual Molecular Dynamics (VMD) software. The two 18-mer segments were then placed in a periodic cubic box. The distance between their atoms and the edge of box was at least 1 nm. Water molecules represented by TIP3P potential were subsequently placed in the box. The side length of the cubic box and the filling water number was 9.00 nm and 23,975 for the GEL3-GLU3 box, 5.66 nm and 5620 for the GEL3-GLU6 box, and 6.40 nm and 8488 for the GEL3-GLU9 box, respectively.

The AMBER99sb-ildnp force field was employed for 18-mer gelatin and glutenin segments in all simulations, which were carried out using GROMACS software. The steepest descent algorithm was employed to minimize the configurational energy of the system. The system was then pre-equilibrated at constant volume for 1 ns, which was then followed by heating the system at constant pressure to 300 K in 1 ns. During heating, the backbones of 18-mer were constrained with a spring constant of 1000 kJ/mol/nm. Lernnard–Jones potential with a cutoff at 1.2 nm and full electrostatics with particle-mesh Ewald summation were employed. Covalent bonds were constrained by the LINCS algorithm with a time step of 2 fs. Production molecular dynamics (MD) simulation was run with a timestep of 2 fs up to 150 ns. A leap frog algorithm for the integration of Newton’s equation was used. The conformations at different times were visualized using the VMD software. The radius of gyration of the 18-mer gelatin–glutenin complex was calculated for every 0.1 ns during the simulation. The binding free energy of the complex was evaluated for every 0.1 ns using the g_mmpbsa program (https://rashmikumari.github.io/g_mmpbsa/) the details of which are given elsewhere (Kumari, Kumar, & Lynn, 2014). The time average for binding free energy only when present as a complex was also calculated from 120 to 150 ns in all the simulations.

### Preparation procedure and the specific volume of bread

The dough was split into three pieces (150 g), pressed by a dough sheeter, shaped into a roll manually, and then put into a bread pan (length × wide × height, 185 × 97 × 54 mm^3^) without the box cover. The dough was fermented at 37 °C and 80% relative humidity for 70 min in a proofer (Tongheng Brand, Foshan, Guangdong, China) and immediately baked in a preheated oven (Leidun Mechanical Co., Ltd, Wuxi, Jiangsu, China), the temperature of which was 210 °C at the bottom and 190 °C at the top for 15 min. At least three loaves of the bread were cooled for 1 h prior to quality evaluation within 4 h at room temperature (25 °C). The other loaves (12–15) of the bread were sealed in a plastic bag and stored at 4 °C (to avoid the fast growth of microorganisms) at a period of 144 h for staling analysis.

After a cooling period of 1 h, the weight of the bread (g) was recorded and the loaf volume (cm^3^) was measured by rapeseed displacement (AACC, 2000). The specific volume (cm^3^/g) of the bread was calculated as the ratio of loaf volume (cm^3^) to weight (g).

### Color analysis of crust and center crumb

The outer crust at the top of the freshly baked bread was measured by a colorimeter (Model NR110, Shenzhen 3nh Technology Co. Ltd., Shenzhen, China) equipped with a D65 light source and a small pore (diameter of 8 mm). The colorimeter was calibrated by a white ceramic plate (diameter of 20 mm) before its color space was adjusted to the CIE-L*a*b* system for L* (ranging from 0 to 100, black to white color strength), a* (positive to negative values, and red to green color strength), and b* (positive to negative values, yellow to blue color strength). The color difference (ΔE*) was calculated as follows:

$$\Delta \text{E}*=\sqrt[{2}]{{\left(\Delta \text{L}*\right)}^{2}+{\left(\Delta \text{a}*\right)}^{2}+{\left(\Delta \text{b}*\right)}^{2}}$$

where ΔL*, Δa*, and Δb* were the differences for L*, a*, and b* between the sample and the reference data. For the analysis of the color of the bread crust, the reference color was from the bread without the addition of gelatin, indicated as bread-0.0% sample.

For the analysis of center crumb (CC) color during storage, the bread was cut to obtain several uniform slices of 12.5-mm thickness with a slicer (Sinmag Mechanical Co., Ltd., Wuxi, Jiangsu, China) at 2, 48, 96, 144 h during a storage period of 6 days. Then, the internal CC color was determined. The CC color at 2 h was defined as a reference color when the color difference (ΔE*) at different storage times was calculated.

### Crumb texture image analysis

After the CC color was measured, the middle slice of the bread was captured by an image scanner. For image analysis, a single (30 × 30)-mm^2^ field of view (Fig. 4) was selected in the center of each slice by ImageJ software (National Institutes of Health, Bethesda, MD, US). The cell to total area ratio (%), cell density (cells/cm^2^), and mean cell area (mm^2^) of cells on the slice were calculated (Sang et al., 2020). The experiments were performed at least three times.

### TPA of the CC

After the scanning, the TPA of the CC of the bread was measured using the TA.XT Plus Texture Analyzer with a P/25 probe. Two whole bread slices (total thickness of 25.0 mm) were compressed at the CC part of slice by the probe. The test conditions were set as follows: retest speed, 1.00 mm/s; test speed, 1.70 mm/s; post-test speed, 10.00 mm/s; trigger force, 5 g; and strain, 40%. The maximum force of the first compression was defined as the hardness of the CC. Springiness was measured by the distance of the detected height during the second compression divided by the first compression distance. Cohesiveness was defined as the area of work during the second compression divided by the area of work during the first compression. Resilience was calculated by dividing the upstroke energy of the first compression by the downstroke energy of the first compression.

### Differential scanning calorimetry

Differential scanning calorimetry (DSC) was performed on an X-DSC7000 system (SII NanoTechnology Inc., Chiba-shi, Japan) to investigate the effect of gelatin on the retrogradation enthalpy of the CC after the instrument was calibrated using indium as a standard under a nitrogen (high purity, 99.999%) atmosphere. The CC after 6 days of storage at 4 °C was freeze-dried, and subsequently milled and sifted through a an 80-mesh sieve. A portion (approximately 2–3 mg) was weighed precisely into aluminum pans, and mixed with twofold deionized water using a microsyringe. The pans were hermetically sealed and equilibrated overnight at 4 °C allowing the dried CC to absorb water completely. The sample pan together with an empty reference pan was held isothermally at 20 °C for 5 min and then heated from 20 °C to 90 °C at a rate of 10 °C/min. The DSC analysis was run in triplicate for each of the samples.

### Water content of different parts in the bread and water activity

The water content (%, w/w, based on the wet sample) of the three parts of the bread was determined using an oven at 105 °C. The water content was calculated as a percentage reduction in the total weight of the sample after 24 h of heating. At different storage times (2, 48, 96, and 144 h), the water activity (Aw) of the crumb was measured using a Aw meter (Novasina Ms1 AW, Switzerland) in 60 s. Then, the bread was cut into three parts: CC (diameter: 30 mm), marginal crumb (outer diameter: 80 mm; inner diameter: 30 mm), and crust (Fig. 4).

### Statistical analysis of data

All the experiments were performed at least three times. The data were analyzed using one-way analysis of variance and the means were compared by Duncan’s multiple range tests, using the statistical software SPSS (SPSS Inc., Chicago, IL, US). The probability value of *P* < 0.05 was considered significant.

## Results and discussion

### Rheological properties of the dough

**Fig. 1:**
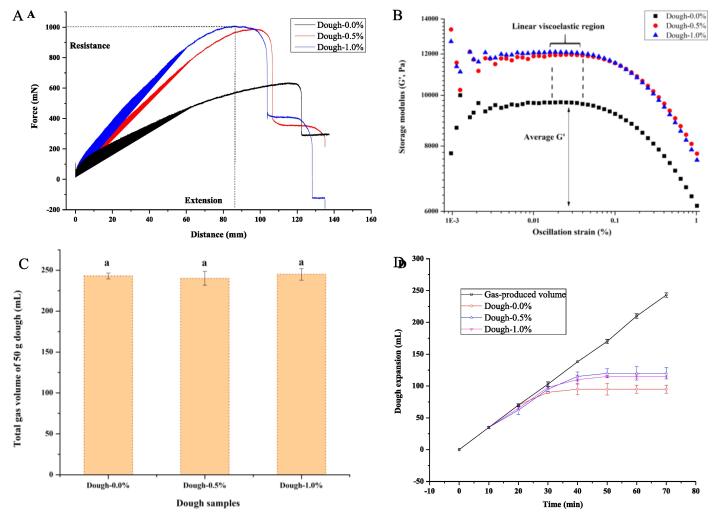
Extension (A) and oscillation shearing (B) properties of dough without yeast, total gas volume (C) and expansion curves (D) of 50-g dough at the different addition levels of fish gelatin (0.0%, 0.5%, 1.0%, w/w, based on wheat flour).

The extension and oscillation shearing curves in Fig. 1A and 1B show that fish gelatin increased the dough strength, e.g. maximum resistance and storage modulus. The rheological parameters of dough (maximum resistance [mN], extension [mm], storage or loss moduli [kPa], and yield stress [Pa]) are summarized in Table S1.

**Fig. 2:**
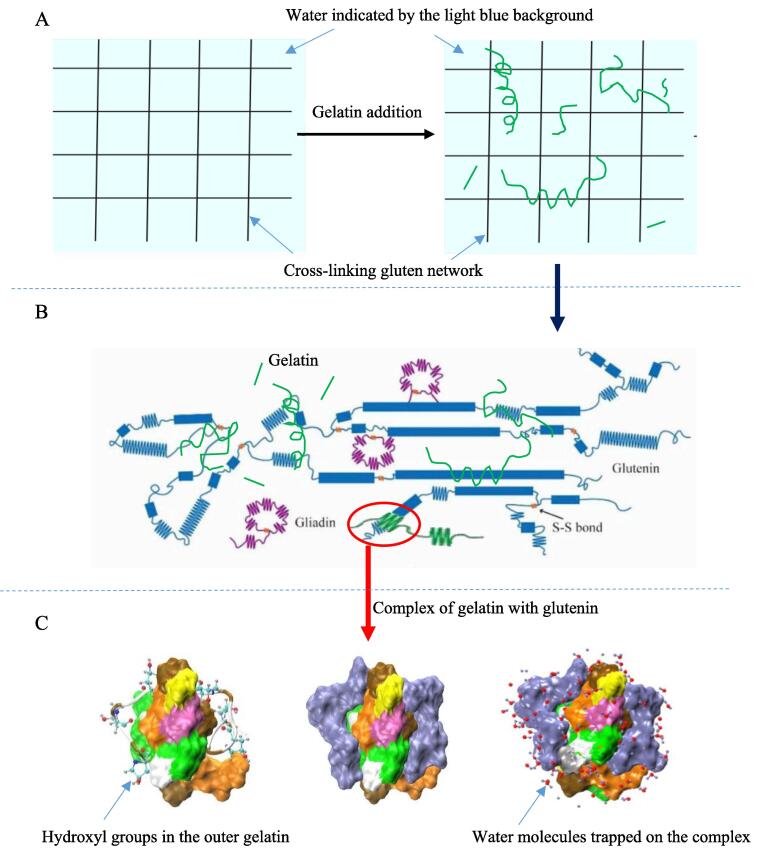
Schematic description of gelatin-induced changes in gluten network (A), glutennin (B), and water mobility on the glutenin surface (C).

**Fig. 3:**
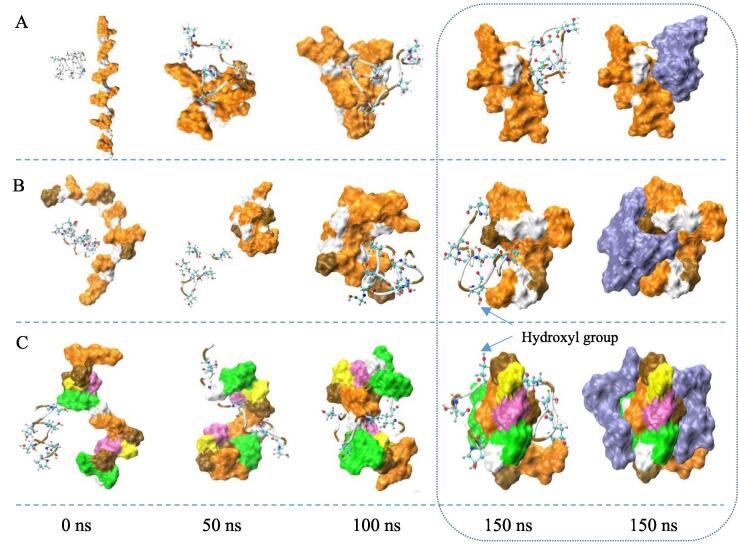
Conformation evolutions during 150 ns of molecular dynamics simulation between gelatin (GEL3, [Gly-Pro-Hyp]_6_) and each glutenin segment consisting of 18 amino acids (A: [GQQ]_6_, B: [PGQGQQ]_3_, C: [GYYPTSPQQ]_2_). Water is not displayed for clarity. The backbone of gelatin segment is drawn as a tube. The hydroxyproline atoms are drawn as color balls, and at the last time of 150 ns the whole gelatin segment is also in ice blue. The amino acids in the glutenin segments are drawn as color surfaces (Glycine [G] is in white; Glutamine [Q] is in orange; Proline [P] is in brown; Tyrosine [Y] is in green; Threonine [T] is in pink; Serine [S] is in yellow.). (For interpretation of the references to color in this figure legend, the reader is referred to the web version of this article.)

The addition of 0.5% or 1.0% gelatin increased the maximum resistance from 652 to 949 and 995 mN compared with the control dough. This result indicated that fish skin gelatin increased the extensional strain hardening effect of dough because of the entanglement of gelatin with glutenin (Fig. 2A and 3). Generally, the extensional strain hardening effect has been shown to be a sensitive indicator of entanglements (Dobraszczyk & Morgenstern, 2003). The same effect was observed that hydrocolloids like λ-carrageenan and pectin that allowed the increase of maximum resistance and the decrease of dough extension, resulting in the strengthening of wheat dough structure (Ribotta et al., 2005). This result was also in alignment with that observed in the dough with the addition of yolk plasma proteins (Sang et al., 2020). However, this result was inconsistent with the conclusion made for the dough with pig skin gelatin, the addition of which did not significantly (*P* > 0.05) change the extensibility and resistance to extension of dough irrespective of the addition amount (Yu et al., 2019). As reported by Yu et al., the pig skin gelatin just altered the water distribution behavior in dough. A possible reason is that pig skin gelatin presents in the form of hydrocolloid particles at room temperature and cannot entangle with glutenin backbone in the dough.

The dough strength is also reflected by the storage modulus (G') in the linear viscoelastic region on an oscillation shearing curve of the dough. Fig. 1B shows that the G' of dough with 0.5% or 1.0% gelatin was higher than that of the control dough in the strain sweep from 0.001% to 1.000% at 1.0 Hz of oscillatory frequency. The G' of dough also represents a cross-linking density of the gluten network in dough. It was inferred that an increase in the cross-linking density was induced by the complexation of fish skin gelatin with glutenin backbone in the network (Fig. 2A). In addition, no significant difference was found between dough-0.05% and dough-1.0% about the G' (*P* > 0.05) as shown in Table S1. This may be due to the saturated capacity for the binding of 0.05% gelatin to glutenin.

The higher loss modulus (G'') and yield stress (σ*) of the dough with fish gelatin (Table S1) indicated that the protein polymers in network moved harder because of stronger cross-linking noncovalent interactions among them after the addition of fish gelatin (Primacella et al., 2018). The value of tanδ is obtained as the G''/G', which indicates the relative contribution of the viscous and elastic components in a viscoelastic material. Table S1 shows that the tanδ values of all dough are<1 at the tested oscillation frequency of 1.0 Hz. This meant that the elastic component dominated over the viscous one in all dough. Tanδ did not change significantly as the addition level of fish skin gelatin increased. Insignificant differences observed in this parameter indicated rather stable contribution of the viscous and elastic components to the rheological behavior of dough when increasing fish skin gelatin level.

The same conclusion was reached when pectin hydrocolloid was added to the gluten model system (Bárcenas, O-Keller, & Rosell, 2009). This may be caused by the formation of complexes between hydrocolloids (Ribotta et al., 2005) and gluten proteins, which could be responsible of increasing equivalent contributions of viscous and elastic components in doughs with higher amount of hydrocolloids. However, the disagreement was, again, observed in pig skin gelatin-involved dough system, where pig skin gelatin induced a progressive decrease in tanδ value as the level of pig skin gelatin increased (Yu et al., 2019). This discrepancy may be ascribed to the poor solubility of pig skin gelatin at room temperature (Karim & Bhat, 2009), which limited the formation of gelatin-glutenin complexes as observed for fish skin gelatin.

### Gas-production and gas-retention properties of the dough

Fig. 1C shows that the addition of fish skin gelatin had no significant effect on the gassing power of the dough, which indirectly reflected that gelatin did not affect the yeast growth in the dough. The measured gas-producing and expansion curves of the dough are shown in Fig. 1D, and the corresponding parameters, including gas-retention volume (mL) and rupture time (min) data, are summarized in Table S1. The addition of 0.5% or 1.0% gelatin can significantly increase the gas-retention volume of a 50-g dough from 95 to 120 mL and extend the rupture time of gas cell walls from 18 to 28 min (*P* < 0.05). This was similar to the observation that yolk plasma lipoproteins improved the expansion property of the dough (Sang et al., 2020). Generally, a stronger gluten network in the dough leads to a higher gas-retention capacity of the dough (Wang, Lee, Xu, & Jin, 2016). Therefore, the gas cell expansion of dough with fish gelatin increased throughout the proofing process as a result of strengthening the gluten matrix network (Song & Zheng, 2007) by the entanglement and aggregation of gelatin with glutenin (Fig. 2, Fig. 3).

**Fig. 4:**
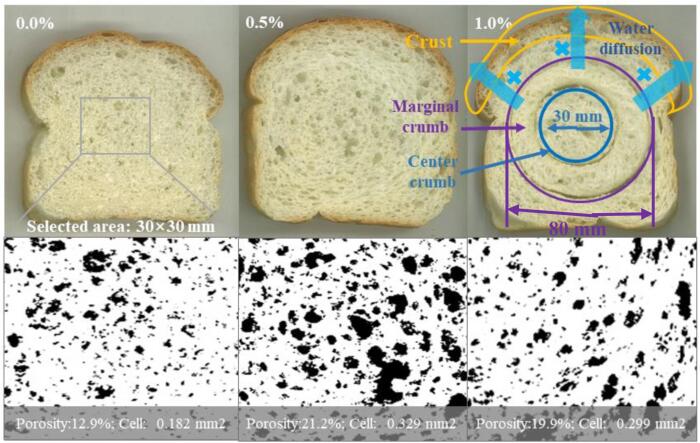
The bread slice profiles and their corresponding threholding images of cell structure of bread with 0%, 0.5%, 1.0% fish skin gelatin, and the water diffusion process through bread is represented at the right corner of figure.

### Interaction between gelatin and glutenin repetitive domains

The gelatin-induced changes in the rheological and gas-retention properties of dough indicated that fish skin gelatin may be able to complex with glutenin (Fig. 2). Furthermore, the water mobility in gluten network might be decreased by the hydroxyl groups on the glutenin-gelatin complex (Fig. 2C), which may affect bread staling. The conformations and binding energy of the gelatin-glutenin complex was further investigated by the molecular dynamics simulation.

Fig. 3A–C show the complexing process of one gelatin segment (GEL3) with different repetitive domains of glutenin (GLU3, GLU6, and GLU9) respectively at different times. The initial distance between the mass center of gelatin and glutenin was maintained the same at 2 nm. Fig. 3 shows that the two segments moved towards each other as time progresses within the first 100 ns because of favorable noncovalent interaction; for example, hydrophobic bonds and van der Waals force. Their conformations unfolded and contacted each other because of their flexibility, while GEL3-GLU9 segments indeed twisted together at last. Most of hydroxyl groups on the gelatin were outside the glutenin-gelatin complex (Fig. 3B and 3C).

The evolution of radius of gyration for gelatin–glutenin complexes for GEL3-GLU3, GEL3-GLU6, GEL3-GLU9 is shown in Figure S1. The radius of gyration represented the compact intensity of complexes. They seemed to stabilize after about 100 ns. In addition, the value of the GEL3-GLU9 complex over 120–150 ns was the lowest, indicating that the GEL3-GLU9 complex had the densest structure. This result was consistent with the lowest binding free energy (Fig. S2). Therefore, the repeating sequence GYYPTSPQQ among the three glutenin repeating motifs was the strongest GEL3-binding site in the repetitive central domain of glutenin.

### Quality of freshly baked bread

Table S2 shows that compared with bread without gelatin, the addition of 0.5% or 1.0% gelatin increased the specific volume of the bread from 4.75 to 5.0 cm^3^/g because of the high gas-retention capacity. This was due to the extension of the cell rupture time, leading to larger cell size (Fig. 4). The mean area per cell in crumb increased from 0.2 to 0.3 mm^2^ (*P* < 0.05), and the porosity increased from 13% to 20%, while the cell density in the crumb did not change significantly (*P* < 0.05), ranging from 60 to70 cells/cm^2^ (Table S2). The crumb porosity (12.9%) was very low because shortening or salt ingredients were not used for the preparation of bread model. Meanwhile, its specific volume and crumb porosity in the research were both lower than the control bread with shortening and salt (Yu et al., 2019). Sang et al. (2020) reported that the yolk-rich dough had larger porosity and cell size, attributing to the lipoprotein-strengthened gluten protein.

Regarding the crust color, the color difference (ΔE*) between the gelatin groups and the control group was approximately 4. The reason may be that the addition of gelatin led to an intensified Maillard reaction (Yu et al., 2019), reflecting by an increase in the yellow value from 29 to 32 (*P* < 0.05). However, gelatin did not significantly affect the whiteness or redness of the crust on the freshly baked bread (*P* < 0.05).

### Effect of gelatin on the staling of crust and CC in the bread

The corresponding mechanism of crust and crumb staling has been attributed to water diffusion from crumb to crust and starch retrogradation taking up water from gluten, respectively (Gray et al., 2003).

**Fig. 5:**
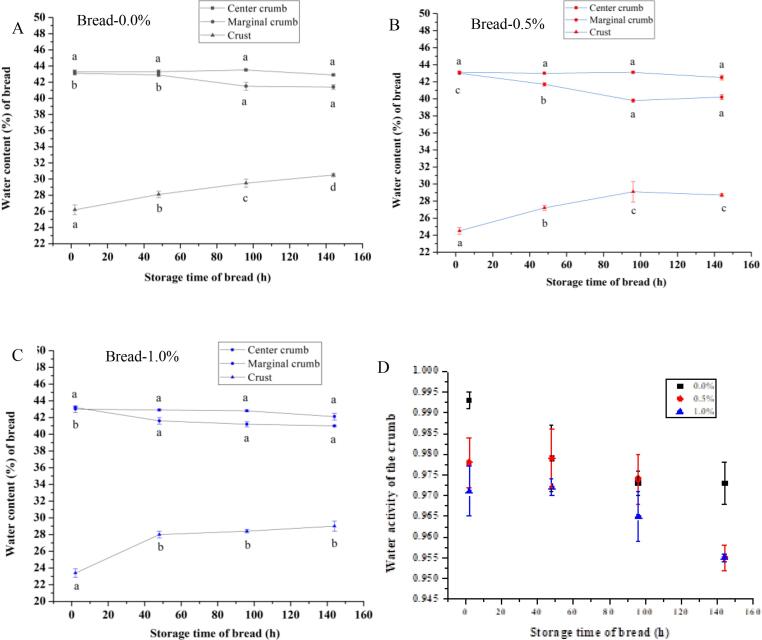
Water content of different parts in the bread with the fish skin gelatin (A-C, 0.0%, 0.5% and 1.0%, w/w, on the wheat flour), and water activity of the crumb (D) at a storage time of 2, 48, 96, and 144 h. The values with different letters in the same curve were significantly different (*P* < 0.05).

As bread aged, a reduction in crispness of crust was usually caused by water diffusion from crumb to crust. As shown in the right slice in Fig. 4, the central slice from bread loaf was divided into three portions from outer to inner bread loaf: crust, marginal crumb and CC. In Fig. 5A, for freshly baked control bread after cooling for 2 h, the crust, marginal crumb, and CC moisture were 26.2%, 43.1%, and 43.3%, respectively. Obviously, the initial moisture gradient was very small for CC and marginal crumb, but large for marginal crumb and crust at the beginning of bread aging. Consequently, the water diffusion would occur from marginal crumb to crust as the control bread aged over 144 h (Fig. 5A). The water content of the marginal crumb decreased from 43.1% to 41.4%, while that of the crust increased from 26.2% to 30.5% (*P* < 0.05). This tendency was consistent with the report by Czuchajowska and Pomeranz (1989). However, regarding breads with 0.5% or 1.0% fish gelatin, the water content of the crust reached the lower equilibrium moisture of 29.1% or 28.0% in a shorter time of 96 or 48 h, respectively (Fig. 5B and 5C). This was attributed to the lower Aw of the crumb in the bread with fish skin gelatin during storage (Fig. 5D), indicating that water molecules were more difficult to escape out from the crumb in the form of water vapor (Yu et al., 2019). The previous molecular simulations (Fig. 2C and Fig. 3C) denoted that a large number of hydroxyl groups on the surface of the hydroxyproline-rich gelatin chain can form hydrogen bonds with water molecules, resulting in the substantial water entrapment in the gluten protein network.

Hardness, water content, and starch retrogradation enthalpy of the CC upon bread staling are summarized in Table 1. The texture of CC in the staled bread at 144 h was too crispy to recover after the first compression in a TPA test; therefore, the hardness was not available at the storage of 144 h. Although the hardness of the control CC (CC-0.0%) increased from 1.37 to 9.38 N over the storage of 96 h (Table 1), the water content (43%) mostly remained unchanged in the absence of water diffusion from the center to marginal crumb. Thus, crumb hardness may be influenced by these additional factors other than water content (Piazza & Masi, 1995). Retrogradation of starch remains the most widely accepted factor related to crumb firming. As shown in Table 1, the enthalpy of starch retrogradation of the control CC increased from 0.29 to 3.46 J/g when the control CC became firm over 96 h.

**Table 1:** The hardness, water content and amylopectin crystalline melting enthalpy and peak temperature of the center crumb (CC) in bread with fish skin gelatin (0.0%, 0.5%, 1.0%, on wheat flour) during storage time of 144 h.

Treatment	Storage time (h)	Hardness (N)	Water content (%)	Enthalpy (J/g)	Peak temperature (°C)
CC-0.0%	2	1.37 ± 0.069 (a, A)	43.3 ± 0.2 (a, A)	0.29 ± 0.01 (a, A)	49.3 ± 1.1 (a, A)
	48	6.36 ± 0.61 (b, A)	43.3 ± 0.2 (a, A)	2.74 ± 0.10 (b, B)	48.2 ± 0.5 (a, A)
	96	9.38 ± 0.40 (c, B)	43.0 ± 0.1 (a, A)	3.46 ± 0.04 (c, C)	49.0 ± 0.3 (a, A)
	144	n.a.	42.9 ± 0.4 (a, A)	3.78 ± 0.08 (d, C)	49.3 ± 0.2 (a, A)
CC-0.5%	2	1.25 ± 0.08 (a, A)	43.1 ± 0.1 (a, A)	0.30 ± 0.02 (a, A)	49.2 ± 0.9 (a, A)
	48	6.00 ± 0.61 (b, A)	43.0 ± 0.0 (a, A)	2.68 ± 0.04 (b, B)	49.5 ± 0.4 (a, B)
	96	6.83 ± 0.66 (b, A)	43.1 ± 0.1 (a, A)	3.26 ± 0.03 (c, B)	49.1 ± 0.3 (a, A)
	144	n.a.	42.8 ± 0.2 (a, A)	3.44 ± 0.10 (d, B)	49.6 ± 0.2 (a, A)
CC-1.0%	2	1.29 ± 0.05 (a, A)	43.0 ± 0.4 (a, A)	0.27 ± 0.03 (a, A)	48.9 ± 1.3 (a, A)
	48	5.60 ± 0.35 (b, A)	42.9 ± 0.2 (a, A)	2.44 ± 0.07 (b, A)	49.0 ± 0.2 (a, B)
	96	6.63 ± 0.23 (c, A)	42.9 ± 0.2 (a, A)	2.80 ± 0.09 (c, A)	49.5 ± 0.4 (a, A)
	144	n.a.	42.8 ± 0.3 (a, A)	3.10 ± 0.11 (d, A)	49.2 ± 0.1 (a, A)

Values with different letters are significantly different (*P* < 0.05) in one column. Lowercase letters are for different storage times by the same treatment, while capital letters are for different treatments at the same storage time. n.a., not available because the staled bread at 144 h was too brittle to be tested.

In Table 1, compared with the control CC, the addition of 0.5% (CC-0.5%) or 1.0% (CC-1.0%) gelatin significantly decreased the hardness and retrogradation enthalpy of CC at the same storage time (*P* < 0.05). Their DSC curves were plot in the Figure S3. Generally, the retrogradation enthalpy of starch represents the melting energy of crystalline amylopectin in retrograded starch. Obviously, starch retrogradation is part of the firming process of the CC although no direct cause-and-effect relationship exists between amylopectin crystallization and CC firming (Gray et al., 2003). However, amylopectin crystallization led to the water shift from gluten to starch upon firming of the CC, as neither water diffusion from crumb to crust nor water evaporation occurred (Bosmans, Lagrain, Ooms, Fierens, & Delcour, 2013). The type-B structure of recrystalline starch has 36 water molecules in the unit cell, whereas only 8 water molecules was observed in the type-A structure of native crystalline starch (Gray et al., 2003).

Therefore, the water content of the CC did not change markedly, but the water redistribution among components (gluten and starch) of the CC still occurred. The complexing of flexible gelatin with gluten backbone (Fig. 3) retarded the water shift from gluten network to starch during the CC firming because of many hydroxyl groups on the surface of the gelatin (Fig. 2C and 3C).

## Conclusions

In this study, the complexing of the fish skin gelatin with the central repetitive domain of the glutenin subunit by noncovalent bonds increased the dough strength and gas-retention capacity, leading to a larger specific volume of the bread. The hydroxyl group of the gelatin in the complex improved the water-holding capacity of the bread. The gelatin hindered not only water diffusion from marginal crumb to crust parts of bread to retard a reduction of crust crispiness, but also water mobility from gluten to starch in the CC to decrease the firming and starch retrogradation rate of the CC. Therefore, fish skin gelatin had a positive effect on the dough property and bread quality as a healthy improver.

## Declaration of Competing Interest

The authors declare that they have no known competing financial interests or personal relationships that could have appeared to influence the work reported in this paper.
