# Supplementary material for: Complexation of fish skin gelatin with glutentin and its effect on the properties of wheat dough and bread
Source: Food Chem X. 2022 Apr 28;14:100319. doi: 10.1016/j.fochx.2022.100319 (PMC9065305; doi:10.1016/j.fochx.2022.100319)
Supplement: Supplementary data 1 [file mmc1.docx]

# Complexation of fish skin gelatin with glutentin and its effect on the properties of wheat dough and bread

**Running Title**: Effect of fish skin gelatin on dough and bread

Shangyuan Sang^1^, Changrong Ou^1^, Yaqian Fu^1^, Xueqian Su^2^, Yamei Jin^3^, Xueming Xu^3,*^

1 Key Laboratory of Animal Protein Food Deep Processing Technology of Zhejiang Province, College of Food and Pharmaceutical Sciences, Ningbo University, Ningbo, Zhejiang, 315832, China.

2 Department of Food Science and Technology, Virginia Polytechnic Institute and State University, Blacksburg, Virginia, 24061, United States.

3 The State Key Laboratory of Food Science and Technology, School of Food Science and Technology, Jiangnan University, Wuxi, Jiangsu, 214122, China.

* **Corresponding author**: Prof. Xueming Xu

**Email:** xmxu@jiangnan.edu.cn

**Address:** School of Food Science and Technology, Jiangnan University, 1800 Lihu Avenue, Wuxi, Jiangsu, 214122, China

| Table S1. Extension, shearing, and expansion properties of developed doughs with fish gelatin (0.0%, 0.5%, 1.0%, w/w, on wheat flour). | | | | | | | | | | |
| --- | --- | --- | --- | --- | --- | --- | --- | --- | --- | --- |
| Treatment | Extension parameters | |  | Oscillation shearing parameters | | | |  | Expansion of 50 g dough with yeast | |
|  | Resistance (mN) | Extension (mm) |  | G' (kPa) | G" (kPa) | tanδ | Yield Stress (σ*, Pa) |  | Gas retention volume (mL) | Rupture time (min) |
| Dough-0.0% | 652±79a | 112±5c |  | 9.61±0.09a | 4.06±0.08a | 0.422±0.005a | 16.9±0.2a |  | 95±5a | 18.2±1.6a |
| Dough-0.5% | 949±31b | 99±5b |  | 12.7±1.0b | 5.40±0.37b | 0.425±0.005a | 24.5±1.9b |  | 120±8b | 28.3±1.7b |
| Dough-1.0% | 995±60b | 86±3a |  | 12.3±0.5b | 5.25±0.28b | 0.428±0.010a | 25.9±3.4b |  | 115±6b | 29.4±2.4b |
| All the experiments were performed in triplicate and data are expressed as average ± standard deviation (n=3). Averages in the same columns with different labels are significantly different with each other (*P* < 0.05). G', storage modulus; G", loss modulus; tanδ (G"/G'), loss factor within the linear viscoelastic region. Yield stress (σ*), the stress where the mean G' within linear viscoelastic region reached a 10% reduction. | | | | | | | | | | |

| Table S2 Specific volume (Vs), crust color, and crumb cell parameters of fresh bread with fish skin gelatin (0.0%, 0.5%, 1.0%, w/w, on wheat flour). | | | | | | | | | |
| --- | --- | --- | --- | --- | --- | --- | --- | --- | --- |
| Treatment | Vs (cm^3^/g) | Crust color | | | |  | Crumb cell parameters | | |
|  |  | White (L*) | Red (a*) | Yellow (b*) | ΔE* |  | Porosity (%) | Cell density (cells/cm^2^) | Mean area per cell (mm^2^) |
| Bread-0.0% | 4.75±0.10a | 43.5±0.6a | 19.4±1.2a | 29.5±0.5a | REF |  | 12.9±0.2a | 70.0±3.3a | 0.182±0.013a |
| Bread-0.5% | 5.05±0.01b | 45.0±1.1a | 21.4±2.6a | 32.1±0.6b | 4.6±0.5a |  | 21.2±3.3b | 64.4±2.1a | 0.329±0.051b |
| Bread-1.0% | 5.01±0.03b | 45.2±0.7a | 19.2±0.5a | 31.9±0.6b | 3.9±0.8a |  | 19.9±1.0b | 66.7±6.3a | 0.299±0.014b |
| Vs, the specific volume (cm^3^/g) of bread. REF indicates that the color of bread crust without gelatin was chosen as a reference color for the calculation of color difference (ΔE*) of crust with gelatin (0.5%, 1.0%). The values were mean±SD from triplicate analyses. Means with different letters in the same column indicate significant difference (*P* < 0.05). | | | | | | | | | |

| Table S3 Texture profile analysis (TPA) and the color of the center crumb in the bread with fish skin gelatin (0.0%, 0.5%, 1.0%, on the wheat flour) upon different storage time at 4°C. | | | | | | | | | |
| --- | --- | --- | --- | --- | --- | --- | --- | --- | --- |
| Treat-ment | Time (h) | TPA parameters | | |  | Center crumb color | | | |
|  |  | Springiness | Cohensiveness | Resilience |  | White (L*) | Red (a*) | Yellow (b*) | ΔE* |
| CC-0.0% | 2 | 0.941±0.011 (b, A) | 0.844±0.001 (c, B) | 0.484±0.004 (c, B) |  | 56.7±1.5 (a, A) | 2.1±0.3 (b, A) | 10.8±0.5 (b, A) | REF |
|  | 48 | 0.884±0.001 (a, A) | 0.426±0.008 (b, A) | 0.154±0.009 (b, A) |  | 62.3±1.0 (b, A) | 0.9±0.1 (a, A) | 9.4±0.2 (a, A) | 5.9±0.9 (a, B) |
|  | 96 | 0.863±0.051 (a, A) | 0.372±0.010 (a, A) | 0.118±0.005 (a, A) |  | 69.1±1.5 (c, B) | 1.0±0.2 (a, A) | 10.7±0.4 (b, A) | 12.4±1.5 (b, C) |
|  | 144 | n.a. | n.a. | n.a. |  | 66.4±0.9 (c, A) | 1.0±0.1 (a, A) | 9.9±0.2 (b, B) | 9.7±0.9 (b, B) |
| CC-0.5% | 2 | 0.928±0.003 (b, A) | 0.843±0.002 (c, B) | 0.475±0.005 (a, B) |  | 61.5±0.5 (a, A) | 2.78±0.17 (b, B) | 10.2±0.2 (c, A) | REF |
|  | 48 | 0.906±0.012 (a, B) | 0.541±0.004 (b, B) | 0.221±0.016 (b, B) |  | 65.2±0.6 (b, B) | 1.00±0.09 (a, A) | 9.6±0.2 (b, A) | 4.2±0.5 (a, A) |
|  | 96 | 0.856±0.043 (a, A) | 0.390±0.037 (a, AB) | 0.134±0.015 (c, B) |  | 63.9±1.2 (b, A) | 1.04±0.04 (a, A) | 10.0±0.1 (c, A) | 3.1±0.9 (a, B) |
|  | 144 | n.a. | n.a. | n.a. |  | 65.0±1.3 (b, A) | 0.84±0.06 (a, A) | 9.0±0.2 (a, A) | 4.2±1.2 (a, A) |
| CC-1.0% | 2 | 0.916±0.024 (b, A) | 0.821±0.005 (c, A) | 0.434±0.009 (a, A) |  | 67.0±1.5 (a, B) | 2.1±0.1 (b, A) | 11.3±0.3 (b, A) | REF |
|  | 48 | 0.879±0.007 (a, A) | 0.585±0.009 (b, C) | 0.233±0.002 (b, B) |  | 66.0±1.1 (a, B) | 0.8±0.1 (a, A) | 9.2±0.8 (a, A) | 2.8±1.0 (a, A) |
|  | 96 | 0.838±0.041 (a, A) | 0.414±0.017 (a, B) | 0.135±0.10 (c, AB) |  | 67.4±0.6 (a, B) | 1.1±0.2 (a, A) | 10.4±0.6 (b, A) | 1.6±0.5 (a, A) |
|  | 144 | n.a. | n.a. | n.a. |  | 67.6±1.0 (a, A) | 0.9±0.1 (a, A) | 9.3±0.2 (a, A) | 2.6±0.4 (a, A) |
| Values with different letters are significantly different (*P* < 0.05) in one column. Lowercase letters are for different storage times in the same treatment, while capital letters are for different treatments at the same storage time. n.a., not available because the staled bread at 144 h was too brittle to be tested. | | | | | | | | | |

**0**

**20**

**40**

**60**

**80**

**100**

**120**

**140**

**160**

**0.8**

**1.0**

**1.2**

**1.4**

**1.6**

**1.8**

**2.0**

**2.2**

**2.4**

**Rg (nm) of glutenin-gelatin complex**

**Time (ns)**

GEL3-GLU3

GEL3-GLU6

GEL3-GLU9

Fig. S1 Radius of gyration versus time (0 to 150 ns) for gelatin-glutenin segment complexes. GEL3, GLU3, GLU6, GLU9 stand for gelatin and glutenin segments consisting of [Gly-Pro-Hyp]_6_, [GQQ]_6_, [PGQGQQ]_3_, [GYYPTSPQQ]_2_, respectively.

Fig. S2 Time average (from 120 to 150 ns) binding free energy for gelatin-glutenin complexes. GEL3, GLU3, GLU6, GLU9 stand for gelatin and glutenin segments consisting of [Gly-Pro-Hyp]_6_, [GQQ]_6_, [PGQGQQ]_3_, [GYYPTSPQQ]_2_, respectively.


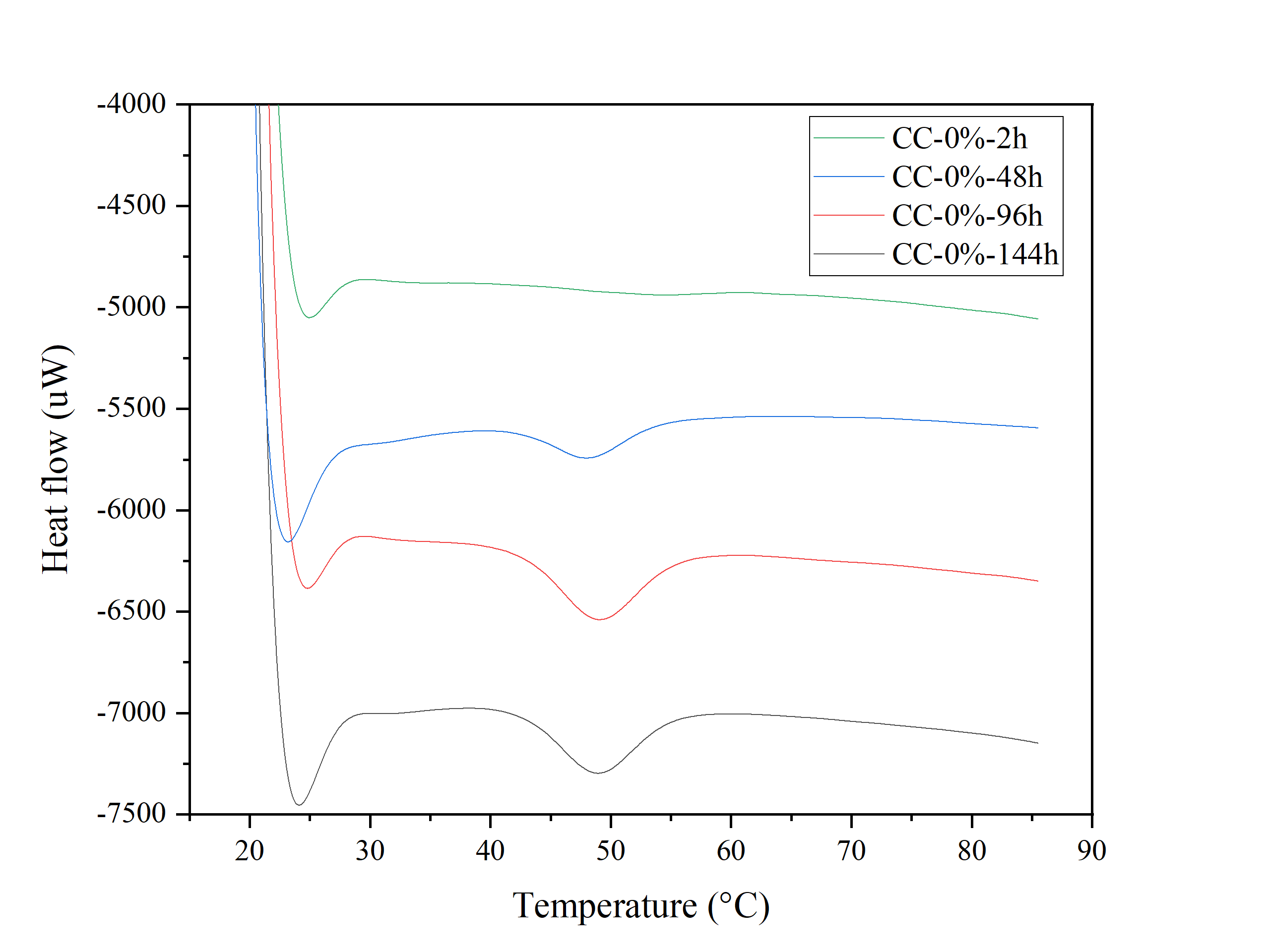


**A**


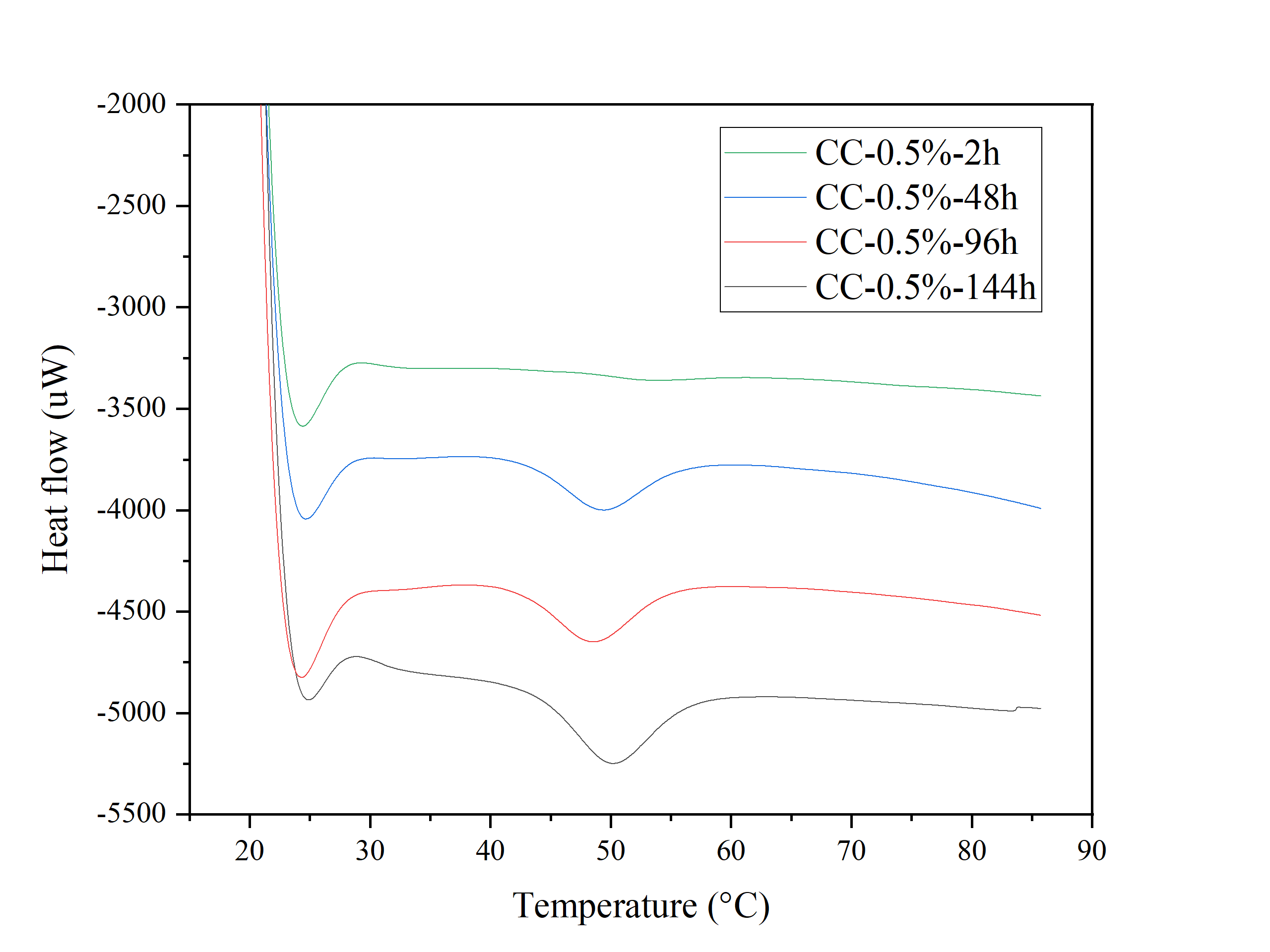


**B**


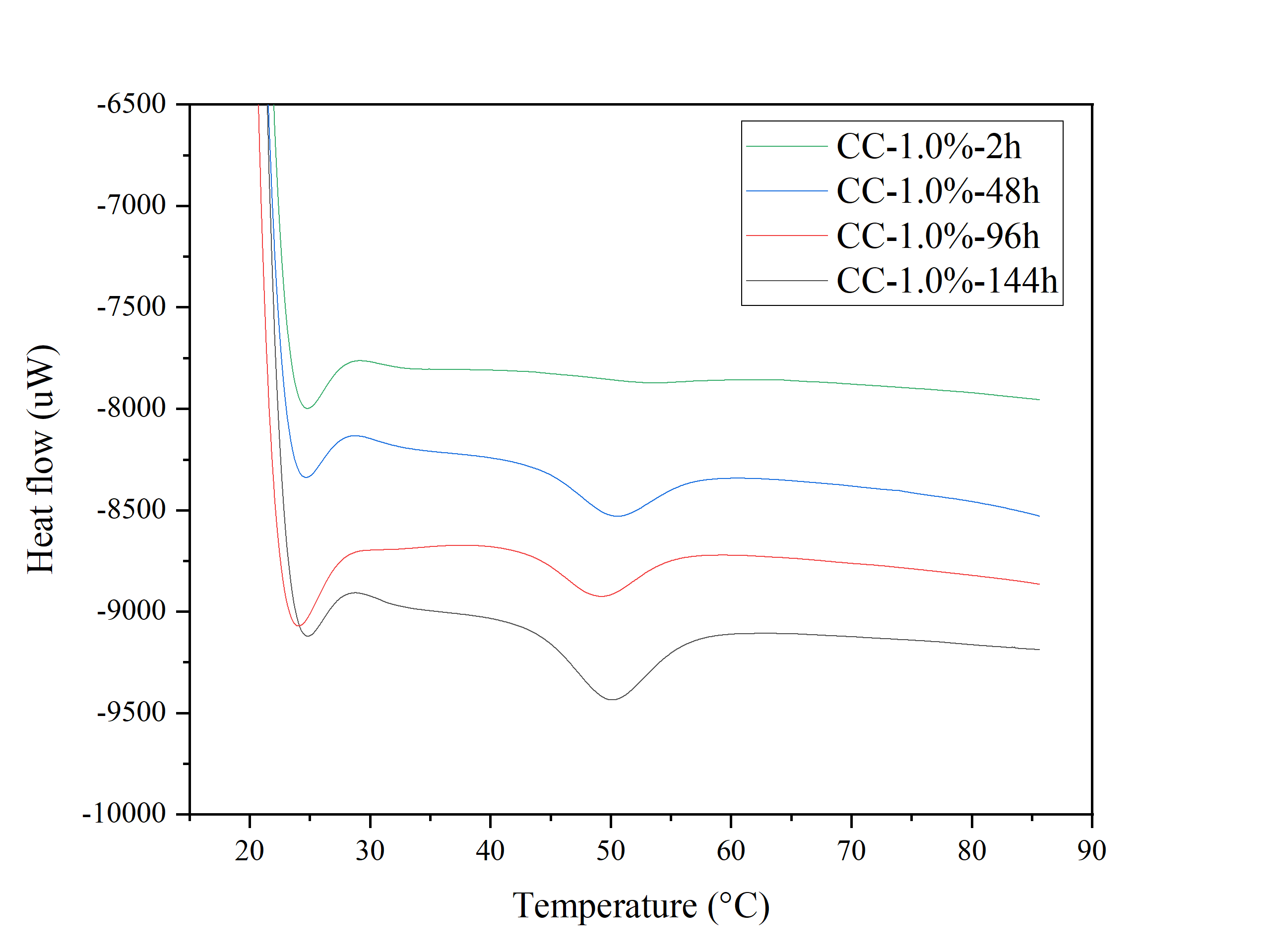


**C**

Fig. S3 The DSC curves of center crumb (CC) during a storage of 2h, 48h, 96h, 144h at different addition levels of fish skin gelatin 0% (A), 0.5% (B), and 1.0% (C).
